# Supplementary material for: RuO2 electronic structure and lattice strain dual engineering for enhanced acidic oxygen evolution reaction performance
Source: Nat Commun. 2022 Jul 1;13:3784. doi: 10.1038/s41467-022-31468-0 (PMC9249734; doi:10.1038/s41467-022-31468-0)
Supplement: Supplementary file 1 — Supplementary Information [file 41467_2022_31468_MOESM1_ESM.pdf]

Supplementary Information

RuO_2 electronic structure and lattice strain dual engineering for enhanced acidic oxygen evolution reaction performance

Yin Qin [1, †], Tingting Yu [1, †], Sihao Deng [2], Xiao-Ye Zhou [3, *], Dongmei Lin [4], Qian Zhang [5], Zeyu Jin [1], Danfeng Zhang [6], Yan-Bing He [6], Hua-Jun Qiu [1, *], Lunhua He [2,7,8], Feiyu Kang [6], Kaikai Li [1, *], Tong-Yi Zhang [9, *]

1. School of Materials Science and Engineering, Harbin Institute of Technology, Shenzhen, 518055, China.
2. Spallation Neutron Source Science Center, Dongguan 523803, China.
3. School of Civil Engineering, Shenzhen University, Shenzhen, Guangdong, 518060, China.
4. Department of Mechanical Engineering, Research Institute for Smart Energy, The Hong Kong Polytechnic University, Hong Kong SAR, China
5. Materials Genome Institute, Shanghai University, 333 Nanchen Road, Shanghai 200444, China.
6. Shenzhen All-Solid-State Lithium Battery Electrolyte Engineering Research Center, Institute of Materials Research (IMR) Tsinghua Shenzhen International Graduate School, Tsinghua University Shenzhen, 518055, China.
7. Beijing National Laboratory for Condensed Matter Physics, Institute of Physics, Chinese Academic of Sciences, Beijing 100190, China.
8. Songshan Lake Materials Laboratory, Dongguan 523808, China.
9. The Hong Kong University of Science and Technology (Guangzhou), Advanced Materials Thrust and Sustainable Energy and Environment Thrust, Nansha, Guangzhou, 511400, Guangdong, China

[*]Corresponding authors: zhangty@shu.edu.cn; likaikai@hit.edu.cn; qiuhuajun@hit.edu.cn; xiaoye_zhou@szu.edu.cn

[†] These authors contributed equally.

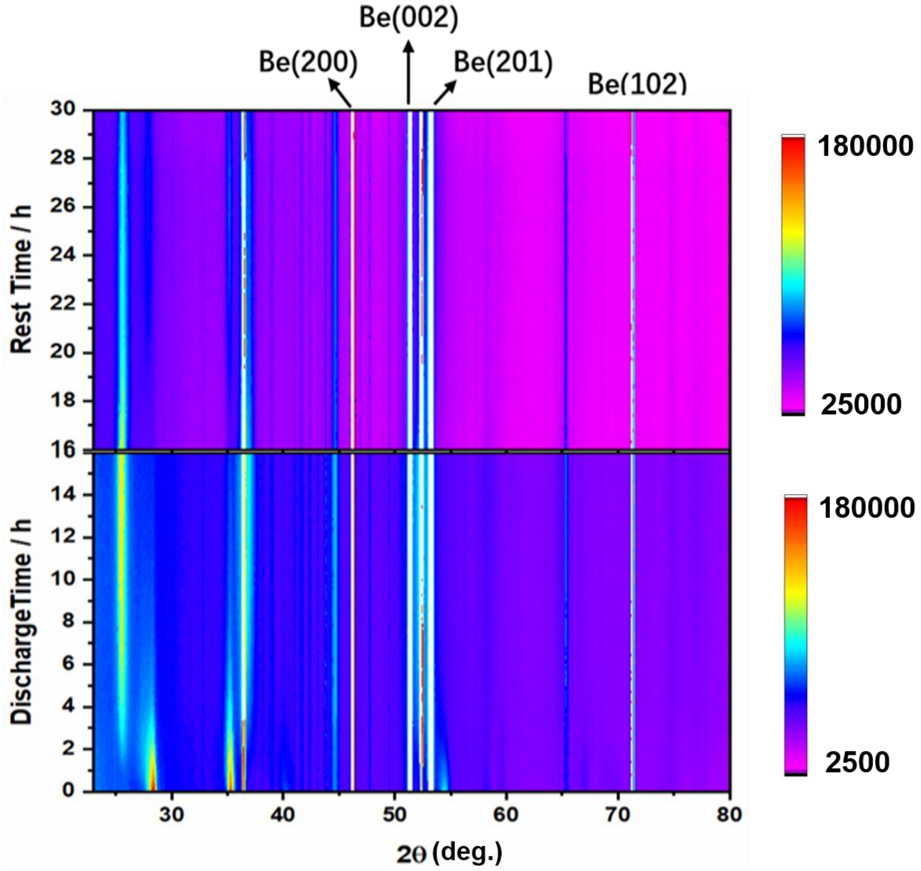

Supplementary Figure 1 | Operando XRD. Operando XRD patterns of RuO_2 during lithiation and rest under a constant current density of 10 mA g^{-1}.

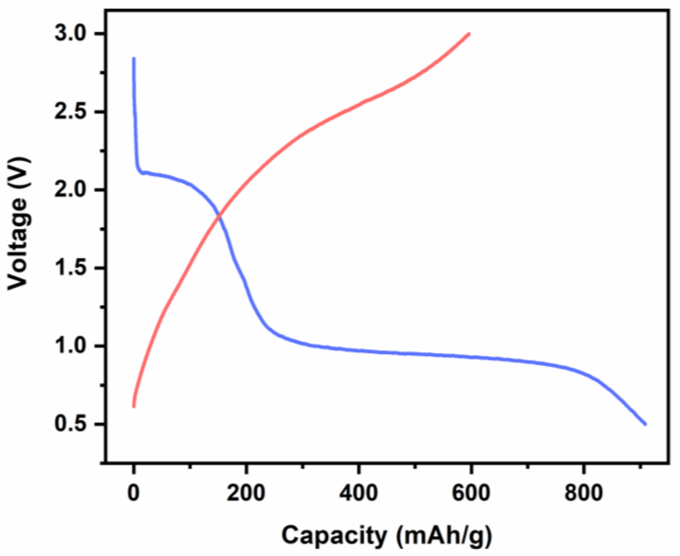

Supplementary Figure 2 | The initial discharge-charge potential profiles. The initial discharge-charge potential profiles of RuO_2 as the working electrode with lithium metal as the counter electrode at a current density of 10 mA g^{-1}. The theoretical specific capacity of RuO_2 is ~ 201 mAh g^{-1}, corresponding to 1 mol Li inserted into 1 mol RuO_2. The coulombic efficiency of the initial cycle is ~ 65%, indicating 35% irreversible capacities occurred during the lithiation. These irreversible capacities are mainly attributed to the side reactions whose lithium ions are not inserted into RuO_2. Here, we assume that the coulombic efficiency is the same when the RuO_2 electrode is cycled between different voltage windows. When the RuO_2 electrode is discharged at a current density of 10 mA g^{-1} for 2h, 9h, 12h, and 16h, the specific capacity is 20, 90, 120, and 160 mAh g^{-1}, respectively. Therefore, the nominal lithium concentration x in Li_xRuO_2 is estimated to be 0.07, 0.29, 0.39, and 0.52.

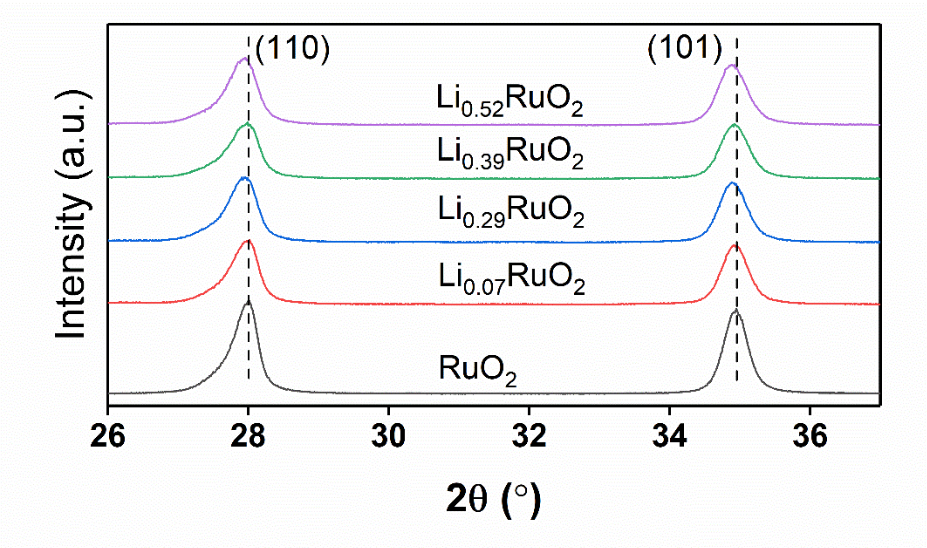

Supplementary Figure 3 | *Ex situ* XRD patterns. Evolution of the (110) and (101) peaks of Li_xRuO_2 with lithium concentration x changing from 0 to 0.52.

Supplementary Note 1:

Determination of the location of Li ions in Li_xRuO_2

Neutron powder diffraction (NPD) patterns of pristine RuO_2 and $Li_{0.52}RuO_2$ were collected on the general purpose powder diffractometer (GPPD) at the China Spallation Neutron Source, and crystal structures were determined by the Rietveld refinement method with the General Structure Analysis System (GSAS)[1]. The results are shown in Supplementary Fig. 4. Obviously, both the pristine RuO_2 and $Li_{0.52}RuO_2$ maintain the rutile RuO_2 structure. Assuming that the Li atoms occupy the Ru sites in $Li_{0.52}RuO_2$, the refinement indicates that the occupancy of Li is only ~ 0.008(3), which means Li atom hardly enters the Ru sites. The NPD patterns of $Li_{0.1}Ru_{0.9}O_2$ and $Li_{0.34}Ru_{0.66}O_2$, in which 10% and 34% Ru cations were replaced by Li cations, were simulated using GSAS program. It is found that the substitution of Ru by Li decreases the intensity of (110) and (211) peaks but has ignorable influence on the (111) and (210) peaks. Comparison of the experimental NPD data of $Li_{0.52}RuO_2$ with the simulated patterns of $Li_{0.1}Ru_{0.9}O_2$ and $Li_{0.34}Ru_{0.66}O_2$ further indicates that the Li atoms didn't replace the Ru cations. Thereby, we conclude that the Li^+ intercalates into the interstice of the RuO_2 lattice. In addition, the background intensity of the NPD pattern of RuO_2 is obviously enhanced after lithium insertion, implying the lattice distortion/strain induced by lithium insertion.

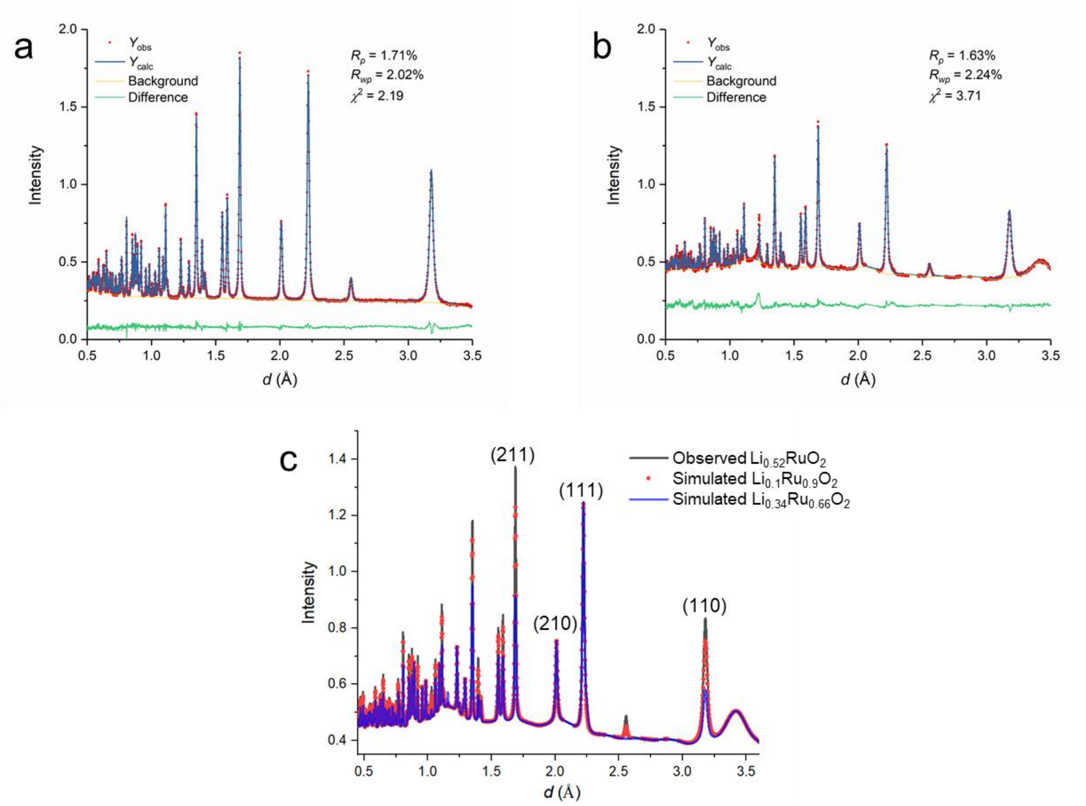

Supplementary Figure 4 | Neutron powder diffraction (NPD) analysis. Rietveld refinement of (a) RuO_2 NPD data and (b) $Li_{0.52}RuO_2$ NPD data. Please note that the peaks originating from the CNT and by-products of the electrochemical process were treated as background and subtracted during the refinement. (c) Comparison of the experimental NPD data of $Li_{0.52}RuO_2$ and simulated NPD patterns of $Li_{0.1}Ru_{0.9}O_2$ and $Li_{0.34}Ru_{0.66}O_2$. In $Li_{0.1}Ru_{0.9}O_2$ and $Li_{0.34}Ru_{0.66}O_2$, 10% and 34% Ru sites are occupied by Li atoms, respectively.

DFT calculations were further performed to investigate the location of Li ions in Li_xRuO_2 from the energy point of view. The energetics of inserting Li into interstitial sites of RuO_2 and replacing the Ru atoms of RuO_2 were calculated, respectively.

Scenario I: Li is inserted into the O-octahedron interstice.

Li atoms were inserted gradually into the O-octahedron interstice in the $Ru_{16}O_{32}$ supercell. For the selection of O-octahedrons for Li insertion, we enumerated all the possible combination and chose the combination with the lowest energy for further

calculation. We then performed structural relaxation of models with Li inserted. The energy cost for Li insertion is calculated by

$$E_{insertion} = E_{Ru_{16}O_{32}+nLi} - E_{Ru_{16}O_{32}} - nE_{Li},$$

where n is the number of Li atoms inserted, $E_{Ru_{16}O_{32}+nLi}$ is the energy of RuO_2 supercell with Li atoms inserted, $E_{Ru_{16}O_{32}}$ is the energy of the RuO_2 supercell and E_{Li} is the energy of a Li atom in vacuum. As more Li atoms are inserted, $E_{insertion}$ becomes more negative, indicating Li insertion is exothermic and spontaneous. $E_{insertion}$ is plotted as a function of n in Supplementary Fig. 5.

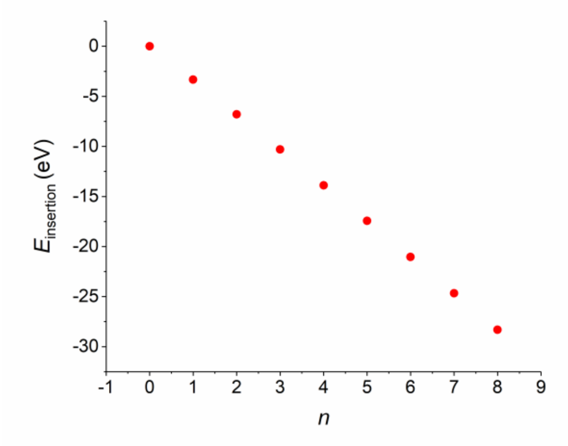

Supplementary Figure 5 | Energetics of inserting Li into interstitial sites. $E_{insertion}$ as a function of n.

Scenario II: Li replaces Ru in RuO_2.

The substitutional energy for replacing Ru by Li in RuO_2 superlattice is calculated as

$$E_{substitution} = E_{Li_nRu_{16-n}O_{32}} - E_{Ru_{16}O_{32}} + nE_{Ru} - nE_{Li},$$

where n is the number of Li atoms to replace Ru atoms, $E_{Li_nRu_{16-n}O_{32}}$ is the energy of RuO_2 supercell with n Ru atoms replaced by n Li, $E_{Ru_{16}O_{32}}$ is the energy of the RuO_2 supercell, E_{Ru} and E_{Li} are the energies of a Ru and Li atom in vacuum, respectively. $E_{substitution}$ is always positive and as n increases, $E_{substitution}$ becomes more positive, indicating that it is energetically unfavorable for Li to replace Ru in RuO_2 superlattice. $E_{substitution}$ is plotted as a function of n in Supplementary Fig. 6.

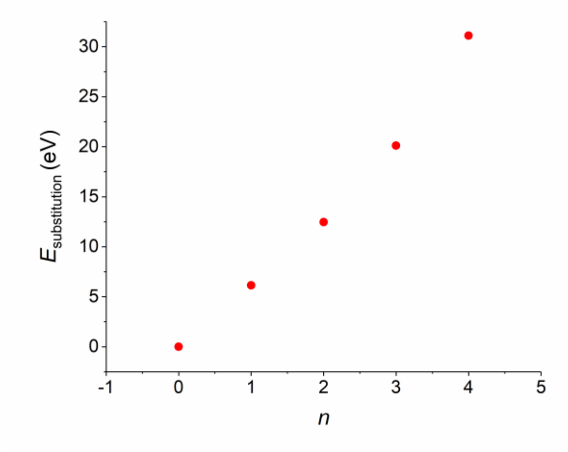

Supplementary Figure 6 | Substitutional energy for replacing Ru by Li. $E_{substitution}$ as a function of n.

From the energy point of view, we can conclude that Li ions tend to insert into the O-octahedron interstice rather than replace the Ru atoms in RuO_2.

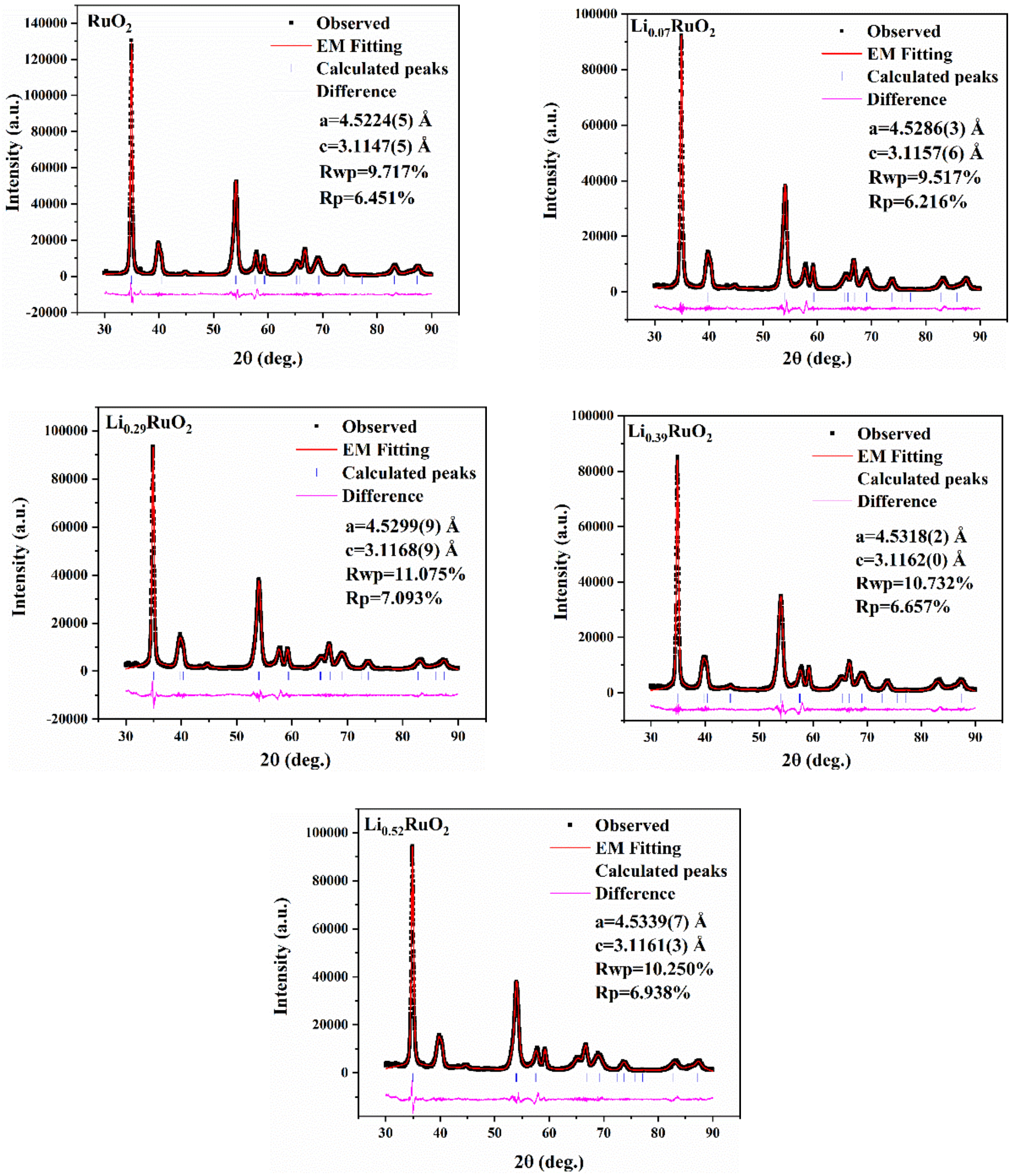

Supplementary Figure 7 | Fitting of the XRD patterns. Fitting of the XRD patterns of Li_xRuO_2 using an Expectation-Maximization (EM) Algorithm based machine learning method, with *x* changing from 0 to 0.52.

Supplementary Table 1 | Lattice parameters of the Li_xRuO_2 obtained by fitting the XRD patterns using Expectation-Maximization (EM) Algorithm based machine learning method.

	R_{wp}	R_p	Lattice constants (Å)	Volume (Å^3)
**RuO_2**	9.717	6.451	a = b = 4.522; c = 3.115	63.705
**$Li_{0.07}RuO_2$**	9.517	6.216	a = b = 4.529; c = 3.116	63.899
**$Li_{0.29}RuO_2$**	11.075	7.093	a = b = 4.530; c = 3.117	63.961
**$Li_{0.39}RuO_2$**	10.732	6.657	a = b = 4.532; c = 3.116	63.999
**$Li_{0.52}RuO_2$**	10.25	6.938	a = b = 4.534; c = 3.116	64.058

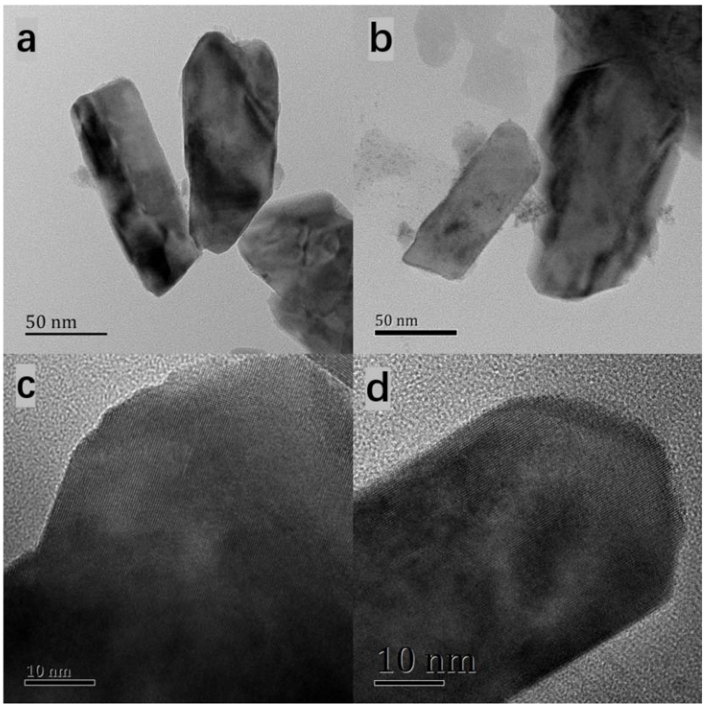

Supplementary Figure 8 | TEM morphology. TEM images of RuO_2 (a, c) before and (b, d) after lithiation for 12 h under a constant current density of 10 mA g^{-1}.

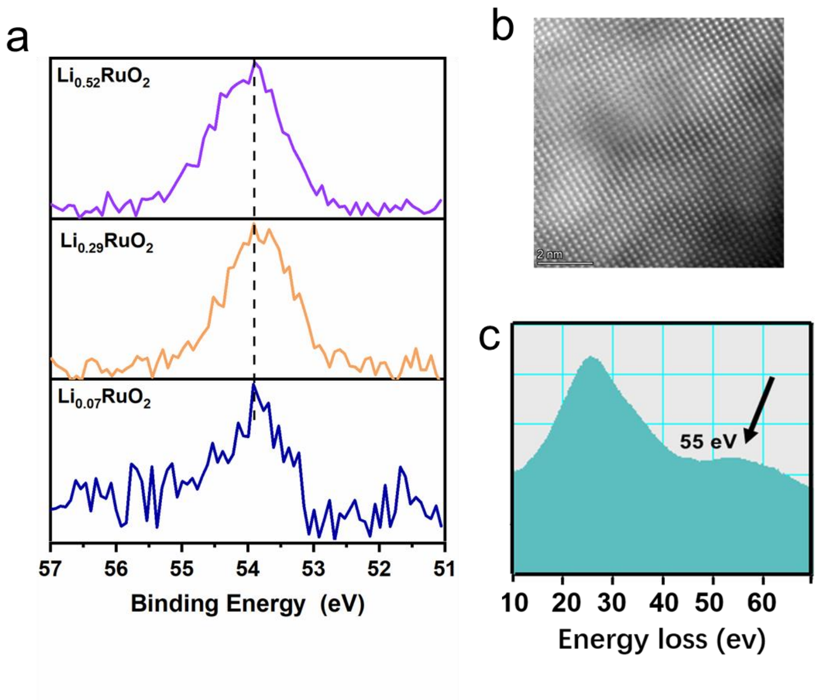

Supplementary Figure 9 | Determination of Li existence. (a) The high-resolution Li 1*s* XPS of Li_xRuO_2. The HAADF-STEM image (b) and Li K-edge STEM-EELS (c) of the $Li_{0.52}RuO_2$.

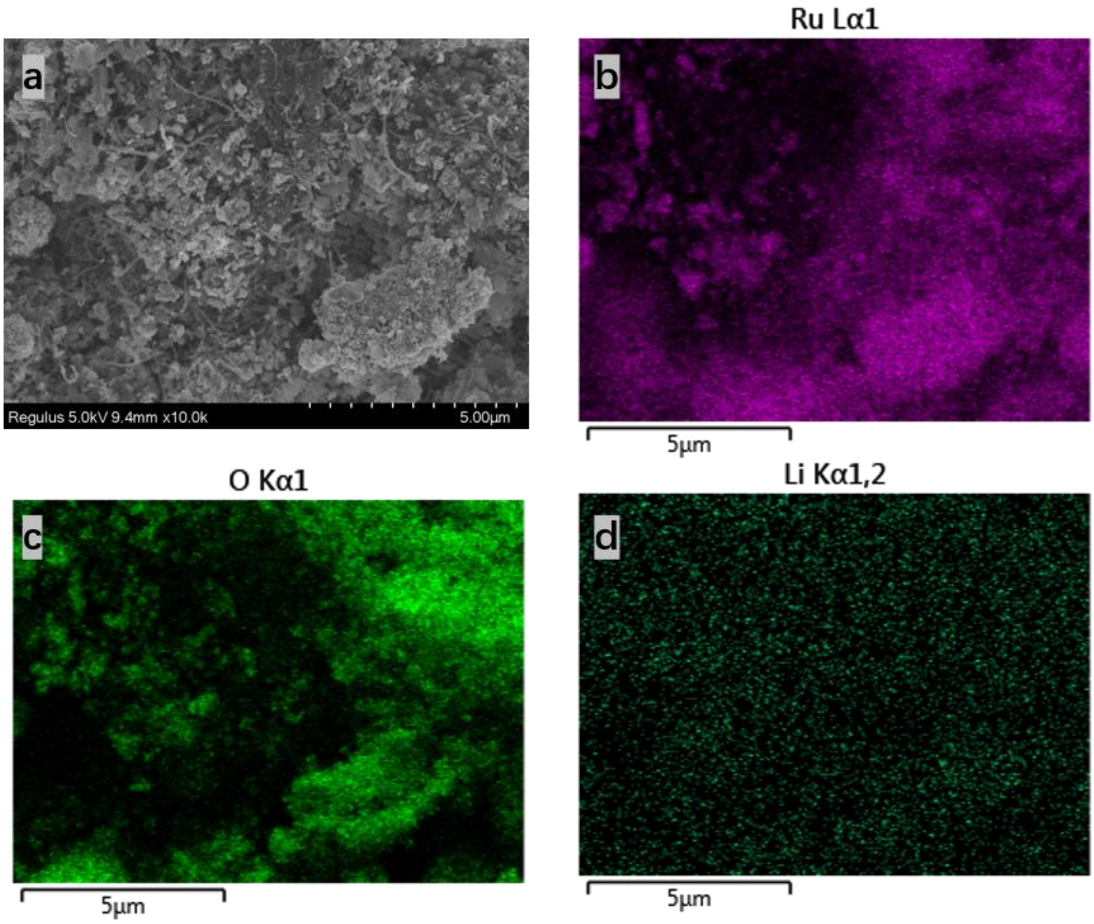

Supplementary Figure 10 | Electrode morphology and composition. SEM image (a) and corresponding EDS mapping (b-d) of the $Li_{0.52}RuO_2$.

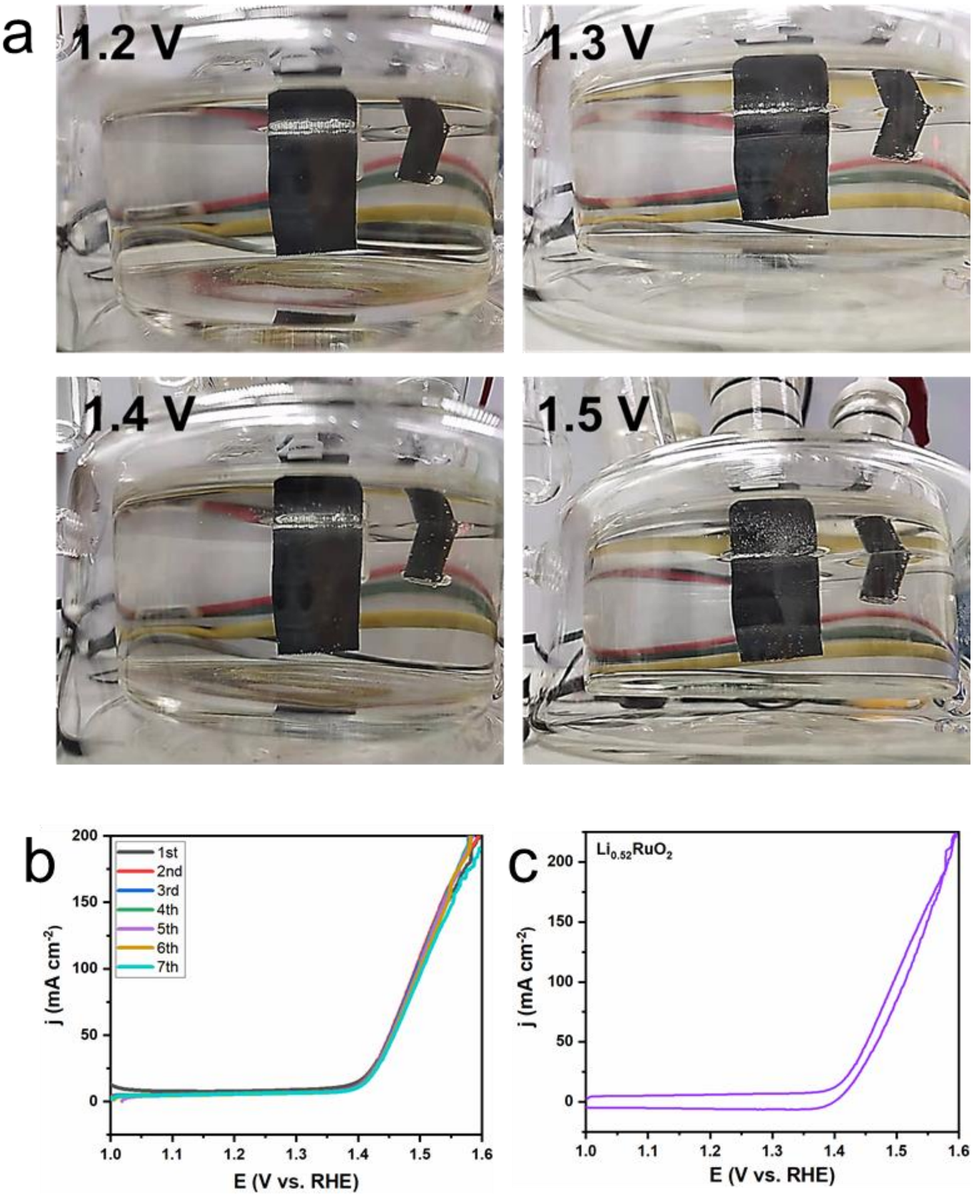

Supplementary Figure 11 | Electrochemical behavior of $Li_{0.52}RuO_2$. (a) Optical images of the $Li_{0.52}RuO_2$ electrode surface when the electrode is under chronovoltometric tests. (b) Polarization curves of $Li_{0.52}RuO_2$ at different cycles. (c) Cyclic voltammetry curves of $Li_{0.52}RuO_2$ in the potential range of 1.0 V – 1.6 V vs. RHE.

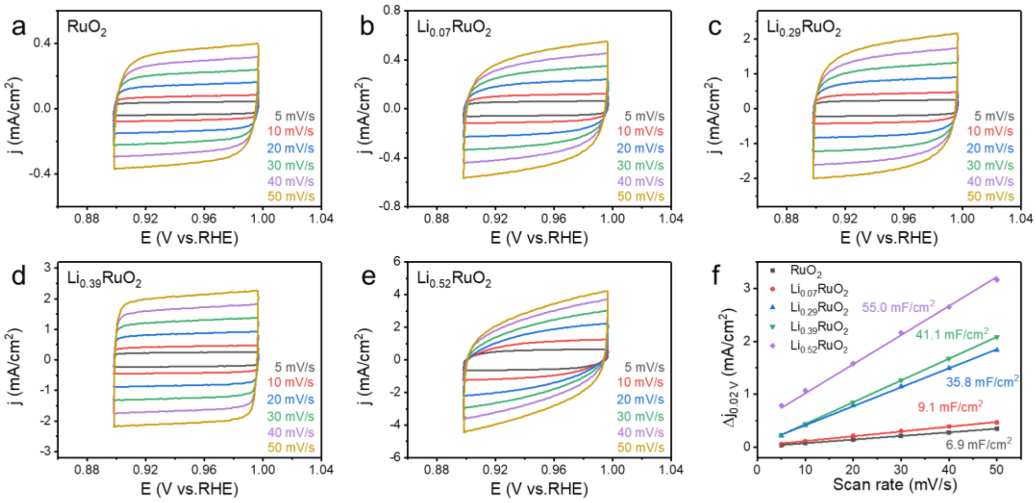

Supplementary Figure 12 | Double-layer capacitance. Cyclic voltammograms of the (a) pristine RuO_2 and the RuO_2 after lithiation (b) $Li_{0.07}RuO_2$, (c) $Li_{0.29}RuO_2$, (d) $Li_{0.39}RuO_2$, and (e) $Li_{0.52}RuO_2$, respectively, collected at scan rates of 5, 10, 20, 30, 40, and 50 mV s^{-1} in 0.5 M H_2SO_4 solution. (f) Capacitive current density plotted against scan rate and linear fitting for the evaluation of C_{DL}.

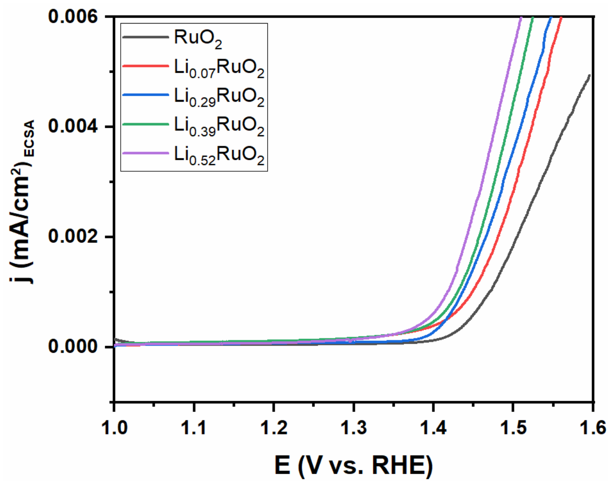

Supplementary Figure 13 | LSV curves. ECSA-based LSVs of the pristine RuO_2 and Li_xRuO_2 with *x* changing from 0.07 to 0.52.

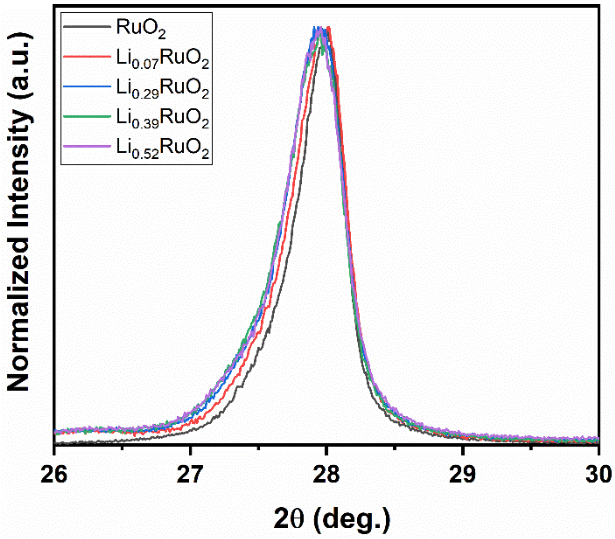

Supplementary Figure 14 | *Ex situ* XRD peaks. The (110) diffraction peak of RuO_2 and Li_xRuO_2 with x changing from 0.07 to 0.52.

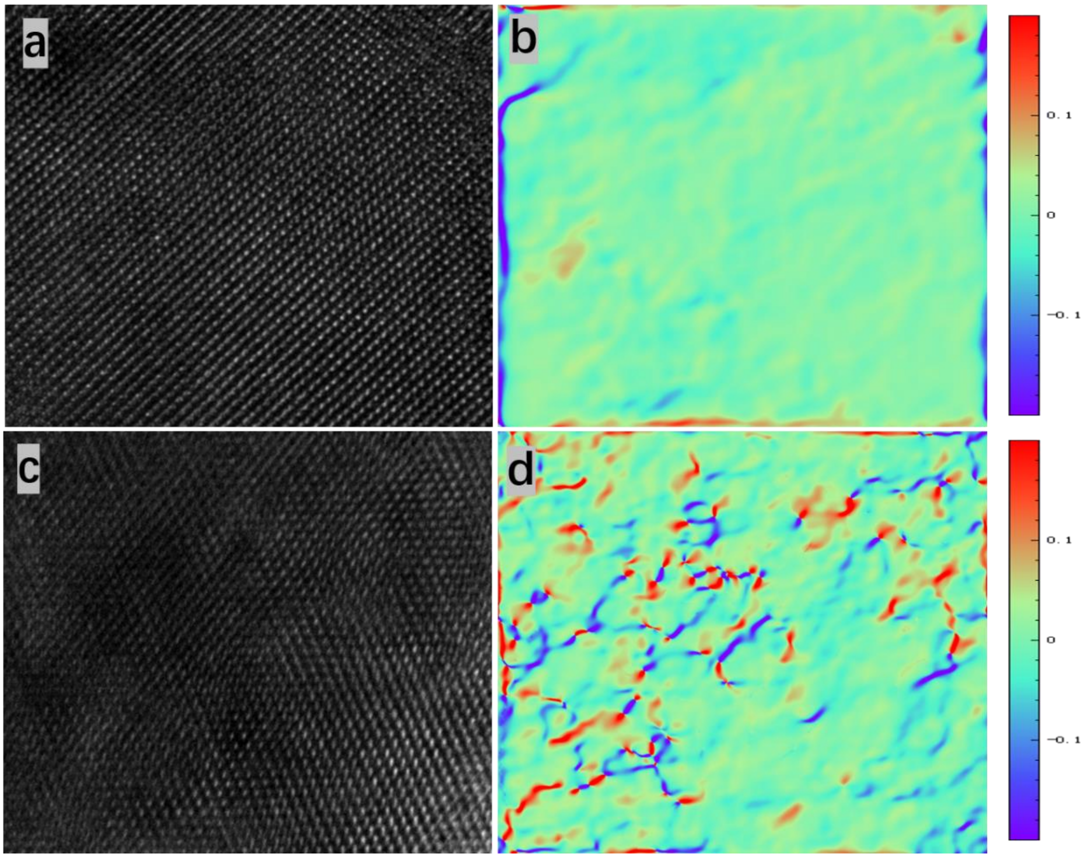

Supplementary Figure 15 | Lattice strain. HRTEM images and corresponding strain (ϵ_{xx}) distribution measured from GPA for (a-b) RuO_2 and (c-d) $Li_{0.39}RuO_2$.

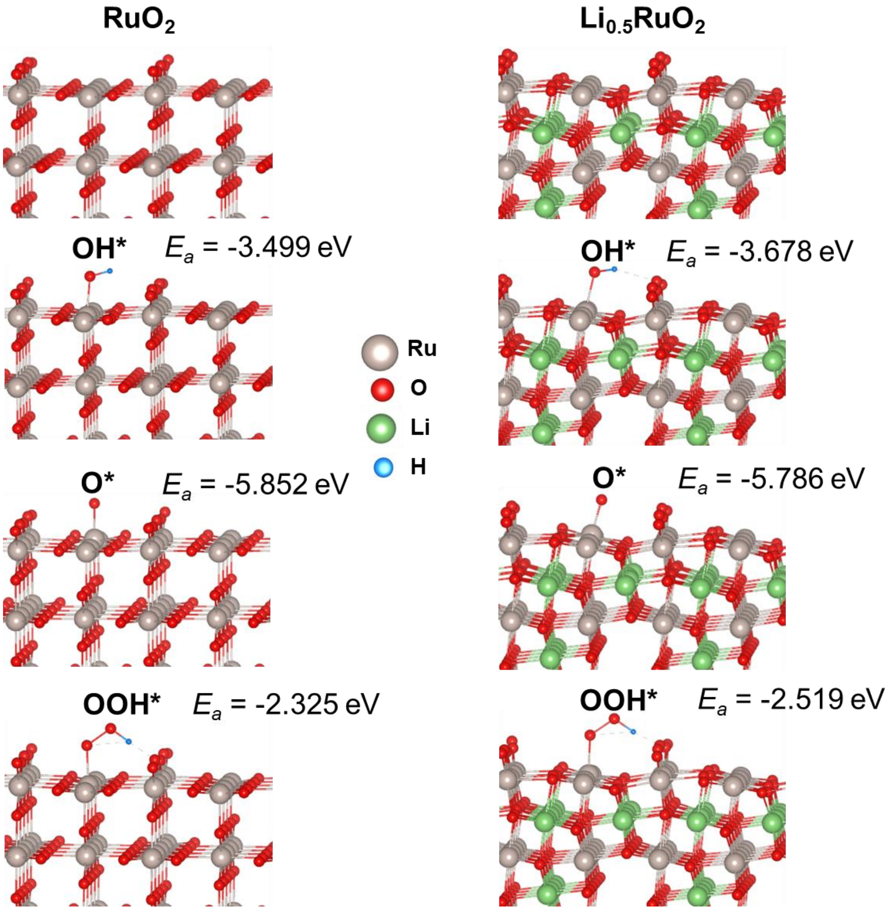

Supplementary Figure 16 | DFT calculations. DFT-optimized structures of RuO_2 and $Li_{0.5}RuO_2$ and adsorption energies (E_a) of the oxo-intermediates.

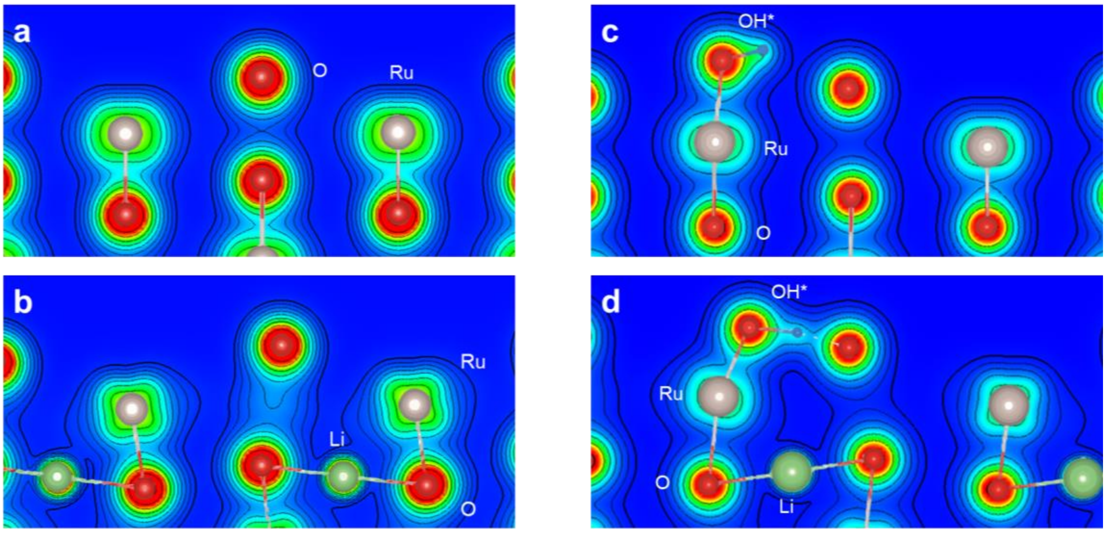

Supplementary Figure 17 | Charge density distribution. The charge density distribution of (a) pristine RuO_2, (b) $Li_{0.5}RuO_2$, and the OH* absorbed on the (110) surface of (c) RuO_2 and (d) $Li_{0.5}RuO_2$. The outermost black curve corresponds to the charge density of 0.0164 $e^-/Bohr^3$.

Supplementary Note 2:

Free energy calculation details:

The Gibbs free energy changes are calculated by the following equations,[2]

$$\Delta G_1 = G(OH^*) + 1/2G(H_2) - G(H_2O) - G_* - eU \quad (1)$$

$$\Delta G_2 = G(O^*) + 1/2G(H_2) - G(OH^*) - eU \quad (2)$$

$$\Delta G_3 = G(OOH^*) + 1/2G(H_2) - G(H_2O) - G(O^*) - eU \quad (3)$$

$$\Delta G_4 = G_* + G(O_2) + 1/2G(H_2) - G(OOH^*) - eU \quad (4)$$

where U is the applied potential taking the normal hydrogen electrode as the reference.

The Bader charge calculation is conducted with the Bader Charge Analysis code. [3, 4, 5] The Bader charge for Ru and Li are calculated as the difference in numbers of valence electrons of Ru and Li atoms as they are in the solid states and as isolated atoms, which demonstrate how many electrons Ru and Li atoms donate (positive Bader charge values) or gain (negative Bader charge values) when bonding with other atoms in RuO_2 or Li_xRuO_2.

Supplementary References:

1. Larson, A. C., Von Dreele, R. B. General Structure Analysis System (GSAS). Technical Report LAUR 86-748, Los Alamos National Laboratory: Los Alamos, NM (2004).
2. Norskov, J. K. et al. Origin of the overpotential for oxygen reduction at a fuel-cell cathode. *J. Phys. Chem. B* **108**, 17886-17892 (2004).
3. Tang, W., Sanville, E., Henkelman, G. A grid-based Bader analysis algorithm without lattice bias. *J. Phys-Condens. Mat.* **21**, 084204 (2009).
4. Sanville, E., Kenny, S. D., Smith, R., Henkelman, G. Improved grid-based algorithm for Bader charge allocation. *J. Comput. Chem.* **28**, 899-908 (2007).
5. Henkelman, G., Arnaldsson, A., Jonsson, H. A fast and robust algorithm for Bader decomposition of charge density. *Comp. Mater. Sci.* **36**, 354-360 (2006).
